# Comparison of Peripheral Biomarkers and Reduction of Stress Response in Patients With Major Depressive Disorders vs. Panic Disorder

**DOI:** 10.3389/fpsyt.2022.842963

**Published:** 2022-03-31

**Authors:** Mi Jin Park, Eun Hye Jang, Ah Young Kim, Hyewon Kim, Hyun Soo Kim, Sangwon Byun, Han Young Yu, Hong Jin Jeon

**Affiliations:** ^1^Department of Psychiatry, Depression Center, Samsung Medical Center, Sungkyunkwan University School of Medicine, Seoul, South Korea; ^2^Bio-Medical Information Technology Convergence Research Division, Electronics and Telecommunications Research Institute, Daejeon, South Korea; ^3^Department of Psychiatry, Hanyang University Hospital, Seoul, South Korea; ^4^Department of Electronics Engineering, Incheon National University, Incheon, South Korea; ^5^Korean Psychological Autopsy Center, Seoul, South Korea; ^6^Department of Health Sciences and Technology, Department of Medical Device Management and Research, and Department of Clinical Research Design and Evaluation, Samsung Advanced Institute for Health Sciences and Technology, Sungkyunkwan University, Seoul, South Korea

**Keywords:** biomarkers, stress response, major depressive disorder, panic disorder, adiponectin

## Abstract

Alteration in stress response seems to affect the development of psychiatric disorders. In this study, we aimed to investigate whether baseline peripheral biomarkers could predict the reduction of stress response among patients with major depressive disorder (MDD) and panic disorder (PD). Patients with MDD (*n* = 41) and PD (*n* = 52) and healthy controls (HC, *n* = 59) were selected and regularly followed up with five visits for 12 weeks. The severity of stress at every visit was assessed using the Stress Response Inventory (SRI), and peripheral biomarkers were measured by blood tests at baseline and 2, 4, 8, and 12 weeks. Interleukin (IL)-6, IL-10, tumor necrosis factor (TNF)-α, interferon (IFN)-γ, C-reactive protein (CRP), adiponectin, and leptin levels were analyzed using enzyme-linked immunosorbent assays. Reduction of stress response was defined as the difference in SRI score between baseline and 12 weeks divided by the baseline score. SRI scores were significantly (*p* < 0.0001) higher in patients with MDD and PD than in HC at every visit after adjusting for variables. In multivariable linear regression, adiponectin levels at baseline were significantly associated with reduction of stress response in patients with PD. When adiponectin increased 1 mg/l, stress response decreased 0.781 points (β = −0.781, S.E. = 0.220, *p* = 0.001). Among the subscales of SRI, somatization had a moderate negative correlation with adiponectin levels (*r* = −0.469). There was no significant association between baseline peripheral biomarkers and reduction of stress response in patients with MDD. Our study showed an inverse association between baseline adiponectin levels and stress response changes in patients with PD, but not in patients with MDD. Thus, differentiated approaches for assessing and treating stress responses of patients with PD and MDD might be helpful. Larger and longitudinal studies are necessary to establish the role and mechanism of action of adiponectin in regulating stress responses in PD.

## Introduction

Stress response is defined as an emotional experience involving physiological, cognitive, and behavioral changes in adaptation to intrinsic and extrinsic stimuli. Stress response involves interworks of the autonomic nervous system, the hypothalamic–pituitary axis, and the immune system, activating both central and peripheral immune cells to release cytokines. Above all, the brain is a key organ that determines behavioral and physiological changes, resulting in atrophic changes in the hippocampus, prefrontal cortex, and amygdala under sustained chronic stressor (1). Appropriate responses of stress systems to a stressor are necessary to maintain homeostasis of the body and sense of wellbeing. However, inappropriate responses to a stressor may disturb the normal growth and development and even cause psychiatric disorders (2). Substantial evidence supports that stress response alteration plays an important role in the development of major depressive disorder (MDD), panic disorder (PD), and post-traumatic stress disorder (3). MDD is a disease with high prevalence (5.1% in females and 3.6% in males), chronic course, and high treatment cost associated with poor social function. It has a very high social disease burden due to decreased productivity (4, 5). MDD is a multifactorial disorder in which adaptation to stress plays an important role in its manifestation. In almost 80% of major depressive episodes, major stressful life events precede within 3 months before the onset of episode. Before major depressive onset, stressors are more than two times more frequent in those with MDD than in healthy controls during the same period (6). As the number of negative events and the severity increase, the risk of developing depression also increases and the onset occurs more rapidly (7). Chronic stressors are associated with poor prognosis, frequent relapse, and poor symptom relief (8, 9). The prevalence of PD is estimated to be 2.7% in 12 months and 4.7% during lifetime according to the National Comorbidity Survey-Replication reports. Its etiology is complex, having interactions with biological factors and environmental factors (10, 11). Panic disorder occurs frequently with other psychopathological comorbidities such as major depression, bipolar illness, and other anxiety disorders. In addition, it is associated with an increased risk of suicide (12). Compared to health controls, PD patients report more life events in the period before onset of PD, and 80% of patients with PD report at least one stressful life event within 6 months before the first panic attack (13, 14).

Since adaptation to stress seems to play an important role in the onset, prognosis, recovery, and even relapse of MDD and PD, the reduction of stress response during the course of the disease can be a good predictor of better prognosis or treatment outcome. There is evidence that an intense acute stressor can stimulate the pro-inflammatory cytokine network, including increased levels of cortisol, interleukin (IL)-6, and tumor necrosis factor (TNF)-α (15). Subjects with psychological stress-induced distress and anxiety show significantly greater increases of interferon (IFN)-γ and decreases in IL-10 than those without such distress or anxiety (16). Leptin and adiponectin are known to have interactions with other stress mechanisms of stress-related disorders (17). Identifying peripheral biomarkers that can predict stress response reduction has useful values in predicting the prognosis and treatment outcome of patients with MDD and PD. However, studies determining the relationship between reduction of stress response and predictive biomarkers are sparse, especially in psychiatric disorders.

Thus, the aim of this study was to compare the Stress Response Inventory (SRI) score at every visit to see any differences by groups. Reduction of stress response was defined as the difference in SRI score between baseline and week 12 (visit 5) divided by the baseline score. We hypothesized that reduction of stress response could be predicted using baseline peripheral biomarkers in patients with MDD and PD.

## Methods

### Participants

**Figure 1:**
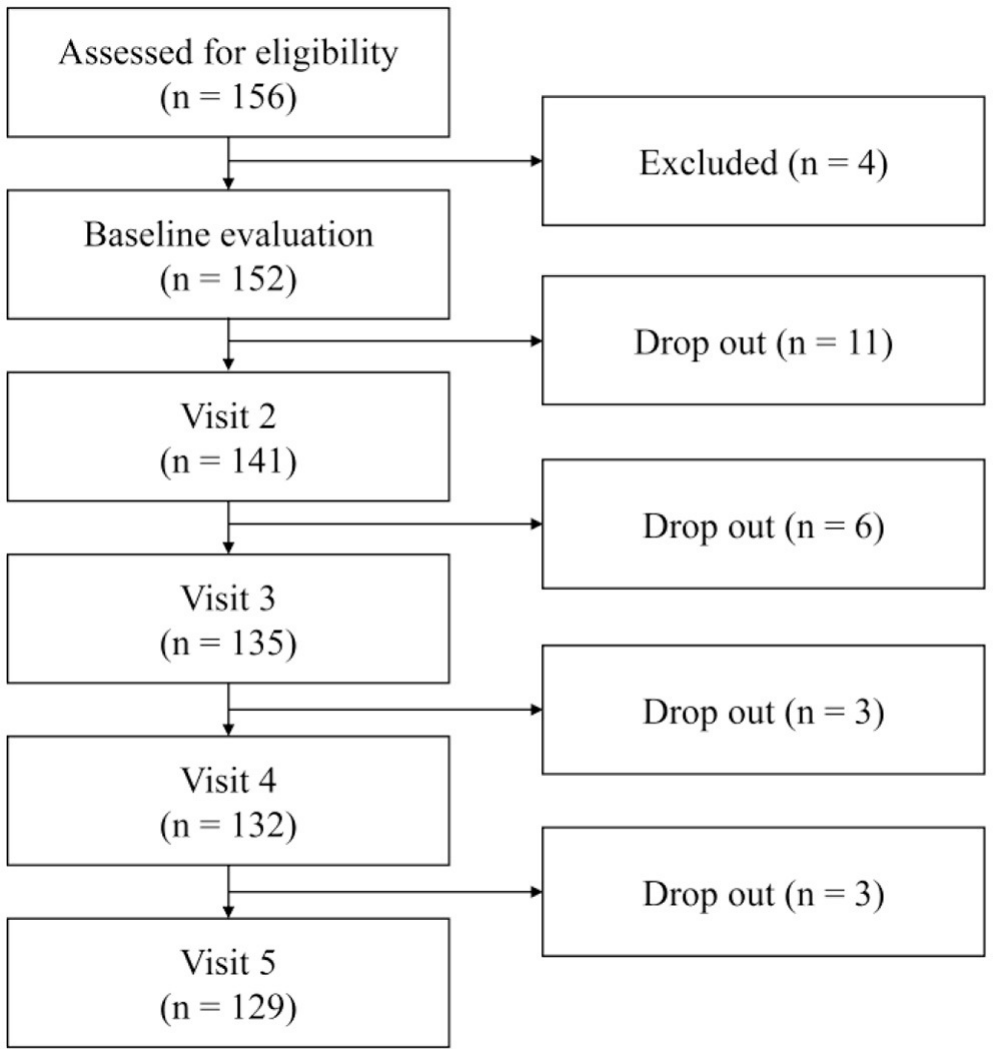
Flowchart of study inclusion from baseline visit to visit 5.

Patients with MDD or PD were recruited from the outpatient clinic of Samsung Medical Center from December 2015 to January 2017. All patients were diagnosed with MDD or PD based on DSM-V diagnostic criteria. Psychiatrists with extensive clinical experience confirmed the eligibility of patients for inclusion in this study after evaluating their psychiatric and medical history. Well-trained psychologists blinded from opinions of psychiatrists evaluated patients' symptoms using the Mini International Neuropsychiatric Interview (MINI) (18). Healthy controls (HC) were recruited *via* advertisements and included in this study after confirming their eligibility using MINI. Exclusion criteria were history of manic or hypomanic episode; schizophrenia; other psychotic disorders; dementia; eating disorders; depressive disorder with psychotic features; intellectual disability; psychiatric disorders with organic causes; substance use disorders; and neurological disorder such as epilepsy, brain damage, and other medically unstable conditions. As shown in Figure 1, out of a total of 156 participants, four were ineligible. After excluding 23 patients who dropped out during consecutive visits, 129 were finally included in this study. All patients maintained pharmacological treatment regimen with selective serotonin reuptake inhibitors (SSRIs), serotonin norepinephrine reuptake inhibitors (SNRIs), norepinephrine dopamine reuptake inhibitors (NDRIs), noradrenergic and specific serotonergic antidepressant (NaSSAs), or tricyclic antidepressants (TCAs) after individualized evaluation. Participants were fully informed of this study and submitted written consent. As neoplastic disease comorbidity, one patient in the MDD group and two patients in the PD group had history of cancer, but the period of diagnosis and treatment was at least 1–10 years apart from this study. As inflammatory disease comorbidity, eight patients in the MDD group and 12 patients in the PD group were reported. This study was approved by the Institutional Review Board (IRB) of Samsung Medical Center (IBR No. 2015-07-151).

### Clinical Assessment

Enrolled participants visited five times for 12 weeks. Sociodemographic information such as age, sex, education years, current smoking, and current alcohol consumption was obtained at baseline clinical evaluation. Measurements of vital signs, BMI, psychiatric evaluation, and blood test were taken at every visit (baseline and 2, 4, 8, and 12 weeks). For evaluating the severity of depression and panic symptoms, Hamilton Rating Scale for Depression (HAM-D) (19) and Panic Disorder Severity Scale (PDSS) (20) were used, respectively.

### Reduction of Stress Response

Stress response was assessed at each visit using the Stress Response Inventory (SRI) invented to evaluate the degree of response to stress through self-report. The SRI consisted of a total 39 questions and seven subscales (tension, aggression, somatization, anger, depression, fatigue, and frustration) to evaluate emotional, physical, cognitive, and behavioral stress responses. Cronbach's alpha for the total scale was 0.97. It was between 0.76 and 0.91 for the seven subscales (21). Each question was evaluated with a 5-point scale ranging from 0 to 4, with a higher score indicating a higher emotional, physical, cognitive, and behavioral stress response. Reduction of stress response was defined as the difference between SRI assessed at baseline and SRI assessed at week 12 divided by the baseline SRI (ΔSRI/SRI at baseline).

### Peripheral Biomarkers

To measure and quantify biomarkers, peripheral blood samples were taken from each participant at each visit. Biomarkers included IL-6, IL-10, TNF-α, IFN-γ, leptin, adiponectin, and C reactive protein (CRP). A total of 50 mg of peripheral blood was drawn from each participant into a serum separator tube and kept at room temperature for 30 min to generate blood clot. After centrifuging at 1,000 × *g* for 15 min, serum samples were collected and stored in a refrigerator under −80°C until assay. All cytokines were quantified using enzyme-linked immunosorbent assays (Human Magnetic Luminex Performance Assay, R&D systems, Minneapolis, MN, USA).

### Statistical Analyses

Categorical data were compared using chi-square test. Continuous data such as baseline demographic and clinical information were compared using analysis of variance. Generalized estimating equation (GEE) with repeated measures was used to evaluate the effects of interaction between two independent variables (groups and visits) on SRI score after adjusting for age, sex, education years, BMI, smoking, and alcohol consumption. Group (MDD, PD, and HC) was considered as a between factor, and time (from baseline to visit 5) was regarded as a within factor. Univariable and multivariable linear regression analyses were performed to determine whether baseline peripheral biomarkers could predict reduction of stress response. All independent and confounding variables were analyzed with a univariable linear regression model to identify the relation between variables and change of stress response. Multivariable analysis was then performed to find predictive peripheral biomarkers for reduction of stress response after adjusting for confounding variables with *p* < 0.05 found in univariable analysis and those with clinical importance. To identify the correlation between proved predictive baseline biomarkers and 12-week changes of SRI subscales (ΔSRI subscale/baseline SRI subscale), Pearson's correlation coefficients were determined. All analyses except GEE were conducted with SPSS version 27 (IBM, Armonk, NY, USA). GEE was executed using SAS version 9.4 (SAS Institute, Cary, NC, USA).

## Results

### Baseline Demographics and Clinical Features

Table 1 shows the results of comparison of baseline information among participants in the three groups (MDD, PD, and HC). The mean age, BMI, proportion of males, and proportion of smokers were not significantly different among the three groups. Subjects in the PD group and the HC group had significantly longer years of education than those in the MDD group (14.53 ± 2.46 years and 14.94 ± 2.43 years vs. 12.90 ± 3.19 years, *p* = 0.001). The proportion of current alcohol consumption was significantly higher in the order of HC, PD, and MDD groups (72.20, 28.85, and 19.51%, respectively, *p* = 0.0001). The HAM-D total score was significantly higher in the MDD group at 18.32 ± 6.06 (*p* = 0.0001). The PDSS total score was significantly higher in the PD group at 3.68 ± 5.76 (*p* = 0.0001).

**Table 1:** Baseline demographics and clinical features by groups (*n* = 152).

	**MDD** **(*n* = 41)**	**PD** **(*n* = 52)**	**HC** **(*n* = 59)**	**Statistics *F* or χ^2^ (*p*-value)**	***post-hoc***
Age (years)^*^	40.95 ± 16.52	41.75 ± 14.08	38.47 ± 14.57	0.72 (0.49)	ns
Sex (M/F)^†^	11/30	20/32	22/37	1.40 (0.49)	ns
Education (years)^*^	12.90 ± 3.19	14.94 ± 2.43	14.53 ± 2.46	7.21 (0.001)	MDD < PD, HC
BMI (kg/m^2^)^*^	22.88 ± 3.47	23.34 ± 3.33	22.80 ± 3.26	0.39 (0.68)	ns
Smokers (%)^†^	7 (17.01)	17 (32.69)	9 (15.25)	5.53 (0.063)	ns
Alcohol consumption (%)^†^	8 (19.51)	15 (28.85)	42 (72.20)	32.00 (0.0001)	HC > PD > MDD
HAM-D^*^	18.32 ± 6.06	13.73 ± 7.58	1.90 ± 1.74	120.70 (0.0001)	MDD > PD > HC
PDSS^*^	3.68 ± 5.76	12.17 ± 5.95	0.02 ± 0.13	100.04 (0.0001)	PD > MDD > HC

*MDD, major depressive disorder; PD, panic disorder; HC, healthy controls; BMI, body mass index; HAM-D, Hamilton Depression Rating score; PDSS, Panic Disorder Severity Scale*.

* *One-way analysis of variance was used; data are given as mean and standard deviation*.

† *Chi-square test was performed for comparative analysis of gender, smoking, and alcohol history*.

### Comparison of SRI Score Across Visits Among the MDD, PD, and HC Groups

Table 2 shows the change pattern of SRI score from visit 1 to 5. The change pattern of SRI across visits was significantly (*p* = 0.0006) different among groups after adjusting for age, sex, years of education, smoking, alcohol consumption, and BMI. As a result of between-group comparison, SRI was significantly higher in both the MDD and PD groups than in the HC group at each visit (all *p* < 0.0001). As a result of within-group comparison, the SRI score was higher at baseline than that at visit 2, 3, 4, or 5 for all groups (all *p* < 0.05).

**Table 2:** Comparison of SRI score at each visit among the three groups (MDD, PD, and HC).

	**MDD** **(*n* = 41)**	**PD** **(*n* = 52)**	**HC** **(*n* = 59)**	**Statistics *F* or χ^2^ (*p*-value)**	***post-hoc***
Visit 1 (baseline)	70.60 ± 34.73	60.65 ± 38.11	14.59 ± 15.07	31.55 (0.0001)	MDD, PD > HC
Visit 2	58.68 ± 40.13.	48.42 ± 42.11	12.73 ± 16.55	16.11 (0.0001)	MDD, PD > HC
Visit 3	46.65 ± 39.14	41.83 ± 40.56	7.75 ± 10.27	12.73 (0.0001)	MDD, PD > HC
Visit 4	48.70 ± 42.08	41.37 ± 42.58	7.59 ± 14.22	11.86 (0.0001)	MDD, PD > HC
Visit 5	40.03 ± 39.73	38.15 ± 41.09	9.66 ± 16.60	7.63 (0.0001)	MDD, PD > HC

*Adjusted for age, sex, education years, BMI, smoking, and alcohol consumption*.

*Bonferroni correction for multiple comparison was applied*.

### Univariable Linear Regression Analyses for Predictive Biomarkers for Reduction of Stress Response in Patients With MDD

In univariable linear regression analysis for MDD patients (Table 3A), alcohol consumption was found to be associated with reduction of stress response among confounding variables. We could not find any significant biomarkers that could predict reduction of stress response in MDD patients.

**Table 3A:** Linear regression on predictors of changes in 12-week stress response intensity using baseline inflammatory markers in patients with MDD and PD.

**Univariable model in patients with MDD (*****n*** **=** **41)**			
	**ΔSRI (SRI at baseline–SRI at week 12)/SRI at baseline**		
	**Beta**	**S.E**.	***p*** **-value**
**Confounding factors**			
Age	−0.003	0.005	0.488
Sex	−0.216	0.166	0.201
Education	0.02	0.024	0.417
Smoking	0.067	0.13	0.611
Alcohol	−0.389	0.147	0.012_*_
BMI	−0.03	0.022	0.174
HAM-D	−0.008	0.013	0.515
PDSS	0.002	0.013	0.881
**Baseline biomarkers**			
IL-6	0.073	0.073	0.324
IL-10	0.05	0.152	0.745
IFN-γ	0.04	0.156	0.798
TNF-α	0.071	0.151	0.642
Leptin	−0.005	0.007	0.52
Adiponectin	−0.001	0.026	0.956
CRP	−0.095	0.076	0.216

*MDD, major depressive disorder; SRI, Stress Response Inventory; ΔSRI, SRI at baseline–SRI at week 12; HAM-D, Hamilton Depression Rating score; PDSS, Panic Disorder Severity Scale*.

* *p < 0.05*.

### Univariable and Multivariable Linear Regression Analyses for Predictive Biomarkers for Reduction of Stress Response in Patients With PD

Tables 3B,C shows univariable and multivariable linear regression analyses for PD patients. In the univariable model, age was significantly associated with reduction of stress response among confounding variables. Adiponectin also had a significant association with reduction of stress response among baseline biomarkers. In the multivariable model, baseline adiponectin was significantly associated with reduction of stress response after adjusting for age and sex with a backward selection method (β = −0.781, S.E. = 0.220, *p* = 0.001). When adiponectin level increased by 1 mg/l, SRI score decreased by 0.781 points.

**Table 3B:** Univariable model in patients with PD (*n* = 59).

	**ΔSRI (SRI at baseline–SRI at week 12)/SRI at baseline**		
	**Beta**	**S.E**.	***p*-value**
**Confounder factors**			
Age	−0.085	0.037	0.024^*^
Sex	−0.955	1.076	0.379
Education	−0.118	0.221	0.594
Smoking	0.424	0.68	0.535
Alcohol	0.617	1.046	0.558
BMI	0.168	0.165	0.314
HAM-D	0.056	0.07	0.429
PDSS	0.079	0.089	0.38
**Baseline biomarkers**			
IL-6	−0.083	0.507	0.871
IL-10	0.992	1.067	0.357
IFN-γ	0.618	1.138	0.59
TNF-α	0.721	1.071	0.504
Leptin	0.039	0.073	0.594
Adiponectin	−0.781	0.220	0.001^*^
CRP	0.053	0.269	0.844

*PD, panic disorder; SRI, Stress Response Inventory; ΔSRI, SRI at baseline–SRI at week 12; HAM-D, Hamilton Depression Rating score; PDSS, Panic Disorder Severity Scale*.

* *p < 0.05*.

**Table 3C:** Multivariable model in patients with PD (*n* = 59).

	**ΔSRI (SRI at baseline–SRI at week 12)/SRI at baseline**		
	**Beta**	**S.E**.	***p*-value**
**Baseline biomarkers**			
IL-6	−0.062	−0.437	0.664
IL-10	0.057	0.400	0.691
IFN-γ	0.058	0.421	0.676
TNF-α	0.189	1.359	0.180
Leptin	0.123	0.870	0.389
Adiponectin	−0.781	0.220	0.001^*^
CRP	0.033	0.235	0.815

*Multivariable model was performed after adjusting for age and sex*.

* *p < 0.05*.

### Correlation Between Baseline Adiponectin and 12-Week Changes in SRI Subscales in PD Patients

We evaluated the clinical correlation between 12-week changes in seven subscales of SRI, and baseline adiponectin level proved as a predictive marker in PD patients (Table 4). Among subscales, tension, aggression, anger, depression, fatigue, and frustration had weak negative correlations with baseline adiponectin. Only somatization had a moderate negative correlation with baseline adiponectin (*r* = −0.469).

**Table 4:** Pearson's correlation coefficients between baseline adiponectin levels and 12-week changes in SRI subscales in patients with PD.

	**Baseline adiponectin**	
	***r***	***p*-value**
Tension	−0.212	0.140
Aggression	−0.078	0.591
Somatization	−0.469	0.001^*^
Anger	−0.075	0.612
Depression	−0.216	0.141
Fatigue	−0.076	0.604
Frustration	−0.013	0.930

*All subscales were calculated as the difference between each SRI subscale score at baseline and week 12 divided by the baseline subscale score*.

Bonferroni correction was performed.

* *p < 0.0071*.

## Discussion

To our best knowledge, this is the first study to identify adiponectin as a predictive peripheral biomarker for stress response reduction in PD patients. The inverse relationship of baseline adiponectin and stress response change was still robust even after adjusting for confounding factors. Biological functions of adiponectin appear to play a more critical role in regulating stress response in PD than other related factors.

Adiponectin is one of the adipocyte-derived peptide hormones synthesized mainly by white adipose tissues. It has anti-inflammatory, insulin-sensitizing, and anti-atherogenic effects (22). Decreased levels of adiponectin are associated with chronic metabolic conditions such as obesity, diabetes, atherosclerosis, cardiovascular disorder, and hypertension (23, 24). A limited but growing body of evidence has suggested the role of adiponectin in various psychiatric disorders including mood disorder, attention deficit hyperactive disorder, post-traumatic stress disorder, obsessive-compulsive disorder, eating disorder, and even neuropsychiatric disorders (25–29). According to a systematic review, adiponectin levels are lower in patients with anxiety, mood, trauma, and stress-related disorders than in healthy controls (30). Studies investigating the role of adiponectin in PD are sparse. One cross-sectional study has compared levels of metabolic parameters and adiponectin in participants with PD and paired healthy controls and found that mean adiponectin levels are significantly lower in the PD group than in healthy controls even after adjusting for BMI and sex (31).

The amygdala is the center for fear processing. It is closely associated with the pathogenesis of panic attack and stress response system. Functional neuroanatomical studies have suggested that the amygdala functions as a center of integrating incoming signals from the peripheral immune system (32). Peripheral cytokines are known to change amygdaloid activity and increase anxiety-like behaviors. TNF-α, IL-6, and CRP are regarded as the main pro-inflammatory cytokines (33). Adiponectin has an inverse relationship with TNF-α and IL-6. It has been demonstrated that adiponectin can exert a variety of anti-inflammatory activities, including inhibition of pro-inflammatory cytokine production and induction of anti-inflammatory factors (34, 35). In addition, adiponectin can cross brain–blood barriers. It appears to be able to directly regulate not only energy and glucose homeostasis but also neurogenesis and synaptic plasticity in various grain regions (36). Recent evidence has suggested that adiponectin can regulate contextual fear extinction in the hippocampus *via* receptor AdipoR2 and modulate dopamine neuronal activity in ventral tegmental area and anxiety-related behaviors through receptor AdipoR1 (37, 38). Taken together, it seems reasonable to speculate that an increase of adiponectin in PD patients might alleviate stress response by reversing the effects of some pro-inflammatory cytokines, activating anti-inflammatory effects, and directly functioning in related brain regions.

Interestingly, only somatization among subscales of SRI had a clinical correlation with adiponectin levels. Some animal studies have suggested an adiponectin-mediated analgesic effect through regulating neuronal activities at the spinal cord level (39, 40). Studies on migraine and osteoarthritis have revealed an association between adiponectin and decreased severity of pain or better treatment response (41, 42). Adiponectin has also been found to regulate norepinephrine and autonomic function through acting on specific nucleus in the hypothalamus. Higher adiponectin levels are associated with better cardiovascular autonomic functions (43, 44). Dysfunction of the autonomic nervous system has been hypothesized to be associated with symptom manifestation and panic attacks in PD. Adiponectin might reinforce the capability of regulating autonomic function and raise the threshold for somatization. As another explanation, adiponectin might go through several stages of action to modulate stress response. Thus, a relatively short period of 3 months might not be long enough for assessing reduction of stress response in domains such as emotion and cognition.

In our study, more than 50% of subjects had values of IL-10, TNF-α, and IFN-γ at zero, which might have limited us to explore their possible correlations with stress response change. When we additionally evaluated non-zero values of these three cytokines in the total study population, baseline IFN-γ levels were negatively associated with 12-week changes in stress response with a correlation coefficient of −0.4266. IFN- γ has been used as a therapeutic modality in malignant tumors for its anti-proliferative and anti-tumor functions. It is also involved in regulating amygdala reactivity to emotional stimuli (45, 46). Although the number of non-zero data was small, findings of a moderate negative correlation (*r* = −0.4266) in this study underlie the possible important role of IFN- γ in stress regulation system.

In patients with MDD, baseline peripheral biomarkers showed no significant associations with stress response changes. For plausible explanations, the high heterogeneity of MDD, sharing inflammation theory as a key mechanism with stress response, and more chronic or add-on impacts on brain structures from both MDD and stress response can be suggested (47, 48). Responses to stressors in MDD characterized by episodic mood changes and those in PD characterized by anxiety reactions might have different brain regions activated in their mechanisms of action and associated biomarkers. Behavioral- and biological-level changes derived from stressors have been suggested to be more obvious when stressors are uncontrollable (49). Characteristics of unexpected panic attacks with anticipatory anxiety in patients with PD reflect such uncontrollability. In MDD, the response to stressors is not simply an anxiety response due to uncontrollability, but rather a wide range of emotional, cognitive, physical, and behavioral changes according to mood changes. Interactions with the hypothalamic–pituitary axis, autonomic nervous system, immune system, and activation of limbic areas seem to be involved in a complex way. Since response patterns to stress are different between MDD and PD, approaches can be differentiated for assessing and dealing with stress responses. In patients with PD, the use of anxiolytics to reduce stress response resulting from uncontrollability might be a good strategy. However, in patients with MDD, the use of antidepressants seems to be a more proper way to deal with overall stress response.

This study is novel in that it reveals baseline adiponectin as a predictor of reduction of stress response in patients with PD. However, it has several limitations. First, this study had a relatively small sample size with a low power to find differences between groups. Applying the results of this study to clinical setting requires validation and replication through larger population-based studies. Second, SRI is focused on stress responses based on the most recent month without providing a start point or duration of stressor. Thus, we could not know whether the stress response was acute or chronic. Also, reduction of stress response was calculated based on a duration of 3 months, which might be too short to reflect changes of stress response. Third, information on medication regimens such as start date and maintenance period of medication was not fully provided. Thus, we could not exclude possible modulating effects of antidepressants or antipsychotics on activities of cytokines or stress responses. Finally, inflammatory diseases may affect cytokine levels. About one-fifth of the MDD group and one-quarter of the PD group were reported to have inflammatory diseases such as hyperlipidemia or diabetes mellitus, but we could not confirm the possible modulating effects because of the relatively small sample size of this study.

In conclusion, greater baseline adiponectin levels predicted more reduction of stress response in patients with PD, but not in those with MDD. It implies that these two highly comorbid disorders, PD and MDD, might have different etiologies in stress response. Thus, differentiated approaches for regulating stress response in PD and MDD might be useful. Further large population-based and longitudinal studies with evaluation of medication and comorbidity are necessary to confirm the role and mechanisms of action of adiponectin in reduction of stress response in PD.

## Data Availability Statement

The raw data supporting the conclusions of this article will be made available by the authors, without undue reservation.

## Ethics Statement

The studies involving human participants were reviewed and approved by the Institutional Review Board (IRB) of Samsung Medical Center (IBR No. 2015-07-151). The patients/participants provided their written informed consent to participate in this study.

## Author Contributions

MP and HJ: conceptualization. EJ, AK, and SB: data curation. HK and HSK: investigation. MP: formal analysis and writing—original draft. HY and HJ: supervision. All authors: writing—review and editing. All authors contributed to the article and approved the submitted version.

## Funding

This work was partly supported by a Grant (No. 2015-0-00062, Development of skin adhesive patches for monitoring and prediction of mental disorders) of the Institute for Information and Communications Technology Promotion (IITP) funded by the Korean government (MSIT) and an Original Technology Research Program for Brain Science through the National Research Foundation of Korea (NRF) funded by the Ministry of Education, Science and Technology (No. NRF-2016M3C7A1947307; HJ). It was also supported by a Grant of the Korea Health Technology R&D Project through the Korea Health Industry Development Institute (KHIDI) funded by the Ministry of Health and Welfare, Republic of Korea (No. HR21C0885).

## Conflict of Interest

The authors declare that the research was conducted in the absence of any commercial or financial relationships that could be construed as a potential conflict of interest.

## Publisher's Note

All claims expressed in this article are solely those of the authors and do not necessarily represent those of their affiliated organizations, or those of the publisher, the editors and the reviewers. Any product that may be evaluated in this article, or claim that may be made by its manufacturer, is not guaranteed or endorsed by the publisher.
